# Editorial: The Alzheimer's Disease Challenge

**DOI:** 10.3389/fnins.2019.00768

**Published:** 2019-07-24

**Authors:** Athanasios Alexiou, Mohammad A. Kamal, Ghulam Md Ashraf

**Affiliations:** ^1^Novel Global Community Educational Foundation, Hebersham, NSW, Australia; ^2^AFNP Med, Wien, Austria; ^3^King Fahd Medical Research Center, King Abdulaziz University, Jeddah, Saudi Arabia; ^4^Enzymoics, Hebersham, NSW, Australia; ^5^Department of Medical Laboratory Technology, Faculty of Applied Medical Sciences, King Abdulaziz University, Jeddah, Saudi Arabia

**Keywords:** AD, amyloid-β, diagnosis, treatment procedures, risk factors, mechanisms

## Biomarkers, Risk Factors, and Pathophysiological Mechanisms of AD

Alzheimer's Disease is a major health challenge with significant social and economic consequences. Ten papers submitted to this Research Topic discuss the biological mechanisms and potential biomarkers for an early and efficient diagnosis, including genetic factors, imaging data, and comorbidities.

Cheng et al. examined the efficacy of certain plasma proteins as potential diagnostic biomarkers of AD using plasma samples from 98 AD patients and 101 healthy elderly controls from Wuxi and Shanghai Mental Health Centers. They discovered that a combination of the plasma proteins BDNF, AGT, IGFBP-2, OPN, cathepsin D, SAP, complement C4, and TTR may provide a diagnostic biomarker for AD in the Chinese population. Even though the sample size of the study was small and various factors can alter plasma protein levels, this plasma panel could provide a solution for the AD population in the future. Additionally, physicians must take into consideration that AD overlaps with other types of dementia, and that multiple comorbidities occur simultaneously. Therefore, a future challenge lies in determining whether this protein panel can exclusively reflect AD, and no other dementia diseases.

Ashraf and Baeesa identified a potentially strong effect of galectin-3 in serum and cerebrospinal fluid (CSF) samples of AD and amyotrophic lateral sclerosis patients, suggesting it as a promising biomarker of these chronic diseases. The authors collected CSF and serum samples from 31 AD patients, 19 amyotrophic lateral sclerosis patients, and 50 normal healthy controls, and performed a comparative analysis of the galectin-3 expression pattern. They also administered a set of neuropsychological assessments that revealed a strong correlation between the galectin-3 levels and cognitive decline in AD patients, and the activation of inflammation and apoptosis, suggesting the necessity of exploring other galectins in relation to neurodegeneration.

Even though functional Magnetic Resonance Imaging (fMRI) is a well-established diagnostic method for every stage of AD development, Bachmann et al. highlight the importance of different methods of graph construction and analysis of fMRI data. Several methods such as the Ward clustering, the Atlas-based clustering, the region growing, and selection algorithm were evaluated in their study based on a sample of 26 healthy controls, 16 subjects with mild cognitive impairment (MCI), and 14 with AD. The results were analyzed with respect to statistical significance of the mean difference in graph properties, the sensitivity of the results to model parameter choices, the relative diagnostic power based on a statistical model, and support vector machines, leading to the conclusion that there are differences between the techniques and their biological interpretations.

Another neuroinformatics study on fMRI data from Bi et al. proposes a new method named the random neural network cluster, which consists of multiple neural networks, to improve the efficient discrimination of AD patients from healthy controls. The authors used the random Elman neural network cluster on the data of 25 AD patients and 36 healthy controls, imported from the AD Neuroimaging Initiative dataset, to select significant features for the identification of abnormal brain regions, to provide accurate AD diagnosis in large samples and high-dimensional data. With an accuracy of >90%, the authors provide the diagnosis of AD by identifying 23 abnormal regions such as the precentral gyrus, the frontal gyrus, and the supplementary motor area, compared to the healthy controls.

Luo et al. evaluated neuroimaging data, including fluorine-18 positron emission tomography—fluorodeoxyglucose of patients with single domain amnestic MCI (sd-aMCI) and multiple domains amnestic MCI (md-aMCI), concluding that the presence of inter-hemispheric connection patterns within the samples. More specifically, the authors evaluated 49 controls, 32 SD-aMCI, and 32 MD-aMCI patients, and presented very promising and clear evidence suggesting inter-hemispheric connectivity as a potential biomarker to monitor disease progression in aMCI patients, simultaneously demonstrating that different damage patterns of inter-hemispheric connectivity may contribute to distinct clinical symptoms in sd-aMCI and md-aMCI.

Although the ε4 variant of apolipoprotein E (ApoE4) gene has already been associated with AD development, Mosca et al. identified a new interaction between the ApoE4 and the RNF219 gene, which correlated with MCI. Here, 83 MCI and 90 AD patients participated, and were initially assessed using neuropsychological evaluations and then genotyped for the APOE and RNF219 polymorphic variants. The authors revealed that AD patients with the ApoE4 and RNF219/G variants presented certain behavioral conditions (including anxiety) that might be useful as potential neuropsychiatric biomarkers.

Sun et al. reported through a single case study the potential pathogenic significance of the SQSTM1 S224X mutation and frontotemporal dementia. The authors described a single case of a 59-year-old woman—with memory decline, mild personality changes, and subtle atrophy of frontal/temporal lobes—in whom an SQSTM1 mutation resulted in the absence of the SQSTM1/p62 protein. Obviously, a larger set of genetic data is necessary to identify the role of this mutation in dementia.

Despite the controversial role of many pathophysiological mechanisms involved in AD development, Frozza et al. highlighted the symptoms and lesions that may affect the patient and that should therefore be considered in drug development approaches in a recent review. While current treatments have a more symptomatic character, the authors described heterogeneous and complex AD signs including mood and behavioral changes, inflammation, and metabolic disturbances, which could lead to the development of hybrid drugs in the search for efficient AD therapy.

Laptinskaya et al. took advantage of the low cost and non-invasive electroencephalography technique to provide a novel and innovative method of neuropsychological assessment for the elderly. In this study 59 participants were classified with subjective memory impairment, 19 as naMCI, and 24 as aMCI, and the authors designed an extended version of the auditory mismatch negativity (MMN) which highly correlated with episodic memory at baseline, and which was underlined, at the 5-year-follow-up, as a potential biomarker for early diagnosis and AD monitoring.

Another research group provided evidence of the potential double role of erythrocyte energy metabolism as a biomarker of impaired cognitive function and as part of new therapeutic procedures (Kosenko et al.). Kosenko et al. discussed the association between the metabolic and antioxidant defense alterations in the circulating erythrocyte population and the neurobiological changes observed in the AD brain, along with the potential usefulness of restoring erythrocyte energy metabolism as an effective treatment.

## Comorbidities and other Related Disorders

Several lesions, symptoms, and comorbidities are commonly present in AD and other related disorders. Six papers featured in this Research Topic discuss common clinical observations and mechanisms between AD and other disorders including GNE myopathy (GNEM), type 2 diabetes mellitus (T2DM), sleep disturbances, hyperlipidemia, and vascular risk. While GNEM is characterized by numerous pathophysiological lesions similar to those of AD, in a detailed review on GNEM and AD-related impairments, Devi et al. draw interesting conclusions on common mechanisms such as the aggregation of amyloid-β (Aβ) and the accumulation of phosphorylated tau and other misfolded proteins. Moreover, in spite of the fact that AD affects mainly brain neurons while GNEM is a rare disease that affects muscle cells, they both share similar disruptions in cellular functions and other common etiologies, including mitochondrial dysfunction, oxidative stress, upregulation of chaperones, and cell death.

In a very detailed review, Chatterjee and Mudher described the strong association and the crossing pathways of T2DM with AD, as a high-risk factor in the elderly population, even though differences can be found between the familial and the sporadic cases. The authors presented novel findings showing that insulin resistance in T2DM can lead to AD-like pathology through abnormal Aβ and tau accumulation, resulting from the loss of synapses, impaired autophagy, and increased neuronal apoptosis.

The correlation between early-onset AD, cognitive impairment, and sleep disorder symptoms are described by Brzecka et al. The authors reviewed evidence from the literature on the influence of low quality sleep, including apneas or disordered breathing, on the circadian fluctuations of the concentrations of Aβ in the interstitial brain fluid and in the CSF related to the glymphatic brain system, which could be associated with cognitive decline and AD pathology.

Ren et al. examined the impact of hyperlipidemia on dementia by applying repetitive transcranial magnetic stimulation (rTMS) on the right dorsolateral prefrontal cortex. The authors observed, in 30 randomly recruited and screened elderly subjects (14 assigned to the rTMS group and 16 to the control group), that rTMS reduces serum lipid levels such as cholesterol and triglycerides, and increases thyroid stimulating hormone and thyroxine levels. Those results show that rTMS may play an effective role in the activity of the hypothalamic-pituitary-thyroid (HPT) axis.

While depression and MCI are both known to contribute to the progression of dementia, Ye et al. reported novel findings in remitted geriatric depression and aMCI patients. In this study, 41 remitted geriatric depression subjects, 51 aMCI subjects, and 64 healthy elderly subjects underwent MRI scans and neuropsychological tests both at baseline and at a 35-month follow-up, and each group was further divided into a declining subgroup and a stable subgroup. Remitted geriatric depression groups with cognitive decline have shown greater vascular risk than the corresponding remitted geriatric depression stable groups, according to the authors, suggesting a relationship with vascular burden. Additionally, compared to the stable aMCI group, the aMCI decline group had a lower left hippocampal volume, which is indicative of neurodegeneration.

Nie et al. focused on the relationships between deep gray matter and loss of brain function due to AD and MCI. Comparing gray subregions of 30 AD patients and 30 MCI patients with 30 normal controls, the authors concluded that even without a decrease in the volume of a gray structure, atrophy might occur in several of its subregions. This atrophy measurement on a subregional level could be characterized as a potential biomarker for early AD diagnosis.

## Treatment Strategies

The rapidly increasing AD population demands holistic solutions and clinical studies with new therapeutic target approaches. Fifteen papers in this Research Topic present innovative promising drugs targeting AD prevention and treatment.

Jan et al. reviewed the failure of the drugs so far approved for treatment in AD, and shift toward a series of drugs to offer a holistic treatment against Aβ deposition and phosphorylated tau. The authors discuss risk factors that affect AD progression, such as mitochondrial dysfunction, endoplasmic reticulum stress and mitophagy, and the potential role of exosomes as efficient drug delivery vehicles for AD therapy.

Another approach involves text mining from traditional Chinese medicine (TCM) database to identify potential drugs and related protein target mechanisms (Meng et al.). Meng et al. retrieved articles from PubMed using AD-related keywords and focused on identifying compounds and proteins involved in AD development. The authors validated their results by applying protein-protein interaction (PPI) networks, western blotting, and co-immunoprecipitation in an AD cell model, in addition to identifying ferulic acid as a potential efficient component of AD treatment.

Menendez-Gonzalez et al. approached the topic of Aβ clearance by designing an alternative method known as the CSF-sink therapeutic strategy for the management of AD, which is based on previously published Aβ-immunotherapies. While Aβ-immunotherapies are directly influencing the CNS, the authors of this review imply that decreases in the concentration of Aβ in the CSF may lead to Aβ removal from the interstitial fluid, and that an increase in the elimination of Aβ by enzymatic degradation may slow down both protein aggregation and AD progression.

In another review study that associates T2DM with AD pathology, Benedict and Grillo presented pieces of evidence that relate the abnormalities in insulin signaling in AD. The authors proposed the existence of common mechanisms in AD pathology and disrupted insulin signaling, and that the pharmacological restoration of normal brain insulin signaling could offer a new therapeutic strategy against AD.

In their review study, Tewari et al. discussed the application of ethnomedicinal methods as dementia novel treatments. The authors evaluated the anti-inflammatory, antioxidative, and antiapoptotic neuroprotective effects of five different plants as potential future drug candidates.

Rahman et al. discussed the molecular signatures of unfolded protein response in elderly subjects, and the resulting neuropathology and memory loss. The role of the endoplasmic reticulum in brain signaling and age-related AD and the neuroprotective effects of modulating the endoplasmic reticulum unfolded protein response in AD are analyzed. Additionally, the authors described the use of astrocytes as potential pharmacological targets in AD therapeutics.

Uddin et al. analyzed the mechanisms and the genes that are related to autophagy and affect AD development. The authors suggested that even though normal autophagy is important for the healthy growth of aged neurons, certain dysfunctions might influence mediators of AD, such as the metabolism of Aβ and tau, the mTOR pathway, neuroinflammation, and the endocannabinoid system.

In a study of two cohorts of older adults, who were recruited independently, Tao et al. examined the effects of Tai Chi Chuan and Baduanjin training on subjects with age-related memory decline. The fractional Amplitude of Low-Frequency Fluctuations in specific frequency bands was proved to provide benefits on memory processes by comparing a Tai Chi Chuan-Baduanjin treatment group with a control group. It is suggested that a 12-week training plan can be used as a therapeutic procedure to improve brain mechanisms.

As microbial infections are a crucial risk factor for neurodegeneration, affecting the vagus nerve, metabolites, neurotransmitters, and immune signaling pathways, Xin et al. investigated the role of oligosaccharides from *Morinda officinalis* (OMO) as a prebiotic that may potentially lead to memory improvement in AD. The authors administered OMO to APP/PS1 transgenic mice and identified potential clinical biomarkers of AD through metabolomics and bioinformatics. Various behavioral experiments revealed that OMO can significantly improve memory in this animal model of AD. The study showed the significance of several metabolites as early indicators of AD development in the animal model, presenting rather strong evidence on the defensive role of OMO on the brain-gut-microbiota axis in AD, providing insights into new potential therapeutic targets for this condition.

Chen et al. provided data from OMO on the brain-gut-microbiota axis of mice to seek for potential drug development for AD. Authors administered OMO to rats with AD-like symptoms. Significant and systematic deterioration were identified in AD-like animals, including in learning and memory abilities, histological changes, production of cytokines, and microbial community shifts, leading to the conclusion of an indirect role of gut microbiota on neurodegeneration. Although microbiota populations that were targeted with OMO were shown to be normally stable and diverse, the authors suggested the use of OMO in future drug designs.

Studies on a mouse model created by Huang et al. analyzed new strategies based on the properties of fluoxetine (FLX) to confront the depression and anxiety of AD subjects. The authors administered FLX via intragastric injection to an APP/tau/PS1 mouse model of AD, suggesting that enabling the Wnt/β-catenin signaling pathway from FLX could decrease amyloidosis in the brain of AD patients. FLX was shown to enhance the protein phosphatase 2A, which then increases β-catenin and inhibits GSK3β activity, both of which affect the Wnt/β-catenin pathway. This leads to the arrest of AD progression and represents a therapeutic formula that should be tested for its potential effectiveness.

Pan et al. tested royal jelly as a potential drug for preventing Aβ activity. Using 24 male white-haired, black-eyed rabbits, which were housed individually under a 12-h light/dark cycle and were provided with food and water *ad libitum*, the authors showed that royal jelly significantly reduces total cholesterol, low density lipoprotein cholesterol, and brain Aβ, lowering the expression of *BACE1* and *RAGE*, while increasing the expression of *LRP-1* and *IDE*. Further, it was noticed that it could be possible to achieve clearance of Aβ, along with an antioxidant impact and positive effects on neuronal metabolism.

Eissa et al. investigated the histamine H3 receptors in relation with AD, experimenting with donepezil on male rats using a passive avoidance paradigm and a novel object recognition task. The authors described the role of H3 receptor antagonists in modulating neurotransmitters and their potential therapeutic perspective.

Bao et al. investigated rat sporadic AD (sAD)—and the differences in pathological conditions in correlation with the sex of the subjects—using a Sprague-Dawley rat model. The findings showed that an intracerebroventricular infusion of streptozotocin results in memory impairment in male but not in female rats. Specifically, there was an increase in the phosphorylation of tau, in Aβ40/42, GSK-3β, and BACE1, with a complete reduction in dendritic and synaptic plasticity in male subjects. Furthermore, male rats showed lower estradiol levels in serum and in the hippocampus than females.

Salmin et al. focused on the application of environmental enrichment (EE) as a potential treatment tool for the efficient management of AD in rats. The authors compared the effects of EE on hippocampal neurogenesis *in vivo* and neurosphere-forming capacity of hippocampal stem/progenitor cells *in vitro*, on a model with AD type of neurodegeneration and a model with physiological brain aging. The authors showed that EE enhances memory signatures related to stem cells in the hippocampus, progenitor cells, and differentiated neurons in young adult rats or in AD model rats and amyloid-based treated rats, but not in the aged rats.

## AD Modeling and Diagnosis

Seven papers in this Research Topic recognized and discussed the problem of the unsuccessful design of drug trials due to the usual late diagnosis of AD and the corresponding irreversible brain alterations.

Ashraf et al. focused on the clearance of Ab plaques by modulating calpastatin signaling, amyloid precursor protein pathways, and Ca^2+^ channels. The authors used stochastic Petri nets to model and simulate these pathways, and identified mechanisms involved in neuronal cell death. The study examined the decrease of plaque production and accumulation by targeting the calpain-calpastatin complexes, and the authors proposed the designing of inhibitors against active calpain using *in silico* methods and *in vitro* experiments.

A study by Sun et al. included experiments on sAD rats treated with streptozotocin. The results showed that different types of hippocampal neural stem cells derived from adult Wistar rats are affected by streptozotocin, and that the expression of metabolism-related genes/proteins is altered in many ways, leading to degenerative disorders.

Khan discussed the necessity of applying complex models for the diagnosis of AD, instead of using a single biomarker. The authors proposed a theoretical preclinical diagnostic algorithm combining heterogeneous evidence, including neuroimaging data, CSF biomarkers, and genetic markers. Additionally, the authors highlighted the diagnostic accuracy, the patient selection in a clinical trial, the universal standardization of diagnostic protocols, the high cost of diagnosis, and the potential ethical challenges as the crucial challenges that must be addressed to apply this algorithm with efficacy in the future.

Tabei et al. compared the effect of non-pharmacological interventions between dementia groups, including physical exercise with music and cognitive stimulation tasks. The authors enrolled 46 patients with mild-to-moderate dementia, 25 of them performed a task consisting of physical exercise with music, and 21 subjects performed cognitive stimulation tasks. It should be noted, however, that the results might be influenced by the limitations of the study, such as the non-inclusion of healthy controls, or the rapid intervention period of the study. The authors concluded that patients with mild-to-moderate dementia, accompanied by cognitive decline and extensive cortical atrophy, did not experience the same improvement after non-pharmaceutical therapy, concluding to the importance of individualized non-pharmacological interventions.

Long et al. discuss the function of MCI assessment as a crucial checkpoint of AD effects. This study enrolled 69 MCI patients and 63 healthy controls. None of the MCI patients had taken any medications that could interfere with cognitive functions. The authors proposed and examined the Hurst exponent of fMRI data from several brain regions, including the left middle frontal gyrus, the right hippocampus, the bilateral parahippocampal gyrus, the bilateral amygdala, the left cingulate gyrus, the left insular gyrus, the left fusiform gyrus, the left superior parietal gyrus, the left orbital gyrus, and the left basal ganglia as proof of MCI identification.

Baig et al. present the role of peptide-based compounds for the diagnosis and treatment of AD in a review study. For the achievement of an early and accurate diagnosis, the authors revealed the importance of peptide-based inhibitors of Aβ, including β-site amyloid precursor protein cleaving enzyme 1, glyceraldehyde-3-phosphate dehydrogenase, tyrosine phosphatase, and the potassium channel KV1.3.

El-Gamal et al. discussed the importance of an efficient early AD diagnosis. The authors designed and programmed a computer-aided diagnosis system for the analysis of Pittsburgh Compound B-Positron Emission Tomography scans, and for the individualized diagnosis based on the analysis of brain regions. The system was validated from an Alzheimer's disease neuroimaging initiative dataset containing 19 normal controls and 65 MCI subjects.

## Conclusion

We hope that this Frontiers Research Topic will enrich the understanding of the AD challenge, with the efforts and commitment of all the authors to whom we give our acknowledgment, and to the reviewers who have contributed by improving and clarifying these diverse contributions through their valuable comments. Finally, we are grateful to the Specialty Chief Editor for Neurodegeneration Prof. Einar M. Sigurdsson for his valuable comments and the Frontiers Editorial team of all the sections for their continuous support.

## Author's Note

The repeated unsuccessful clinical trials on the amyloid-beta targeting medications and the pessimistic calculations of the cost burden of Alzheimer's disease (AD) will have an impact in the next few decades, posing serious challenges for humanity with severe social implications. To date, holistic solution for the disease has yet to be identified, although quite a few alternative diagnostic and treatment procedures have been presented during the past years, considering the latest Alzheimer's classification. The range of scientific fields actively involved in the race for a cure for AD has been impressively extended, allowing the combination of creative ideas using modern technology with innovative therapeutic agents and traditional pharmaceutical practices. In this way, common etiologies and mechanisms in other related disorders or aspects of neurodegeneration could be identified. This Research Topic titled ‘Alzheimer's disease as a current challenge’ offers a contribution of 38 innovative papers on the multidimensional and crucial social problem of AD management. A total of 284 authors presented their different perspectives, research updates and studies in several sections of the Frontiers journals, describing methods on potential diagnosis and treatment.

## Author Contributions

All authors listed have made an equal, substantial, direct and intellectual contribution to the work, and approved it for publication.

### Conflict of Interest Statement

The authors declare that the research was conducted in the absence of any commercial or financial relationships that could be construed as a potential conflict of interest.

